# Risikostratifizierung von Notfällen während der COVID-19-Pandemie in der Zentralen Notaufnahme

**DOI:** 10.1007/s00063-020-00748-2

**Published:** 2020-10-28

**Authors:** M. Wieckenberg, V. Meier, S. Pfeiffer, S. Blaschke

**Affiliations:** 1 Zentrale Notaufnahme, Evangelisches Krankenhaus Göttingen – Weende, Göttingen, Deutschland; 2 Krankenhaushygiene, Evangelisches Krankenhaus Göttingen – Weende, Göttingen, Deutschland; 3 Institut für Medizinische Statistik, Universitätsmedizin Göttingen, Göttingen, Deutschland; 4 Interdisziplinäre Notaufnahme, Universitätsmedizin Göttingen, Robert-Koch Str. 40, 37075 Göttingen, Deutschland

**Keywords:** COVID-19 Pandemie, Zentrale Notaufnahme, Risikokategorien, Hygienemaßnahmen, Infektionsprävention, COVID-19 Pandemic, Emergency Department, Risk categories, Hygienic procedures, Infection prevention

## Abstract

**Hintergrund:**

Die COVID-19-Pandemie stellt für das Personal der Zentralen Notaufnahme (ZNA) eine sehr komplexe Herausforderung dar. Neben der regulären Notfallversorgung ist die frühzeitige Detektion und Isolation von COVID-19-Fällen erforderlich, um eine hausinterne Transmission der Infektion zu verhindern und den Schutz des Personals zu gewährleisten.

**Methoden:**

Es wurde ein Modell zur Risikostratifizierung von SARS-CoV-2-Verdachts- und COVID-19-Fällen mit 5 Risikokategorien (RK) auf Basis der Kriterien des Robert-Koch Instituts (RKI) entwickelt und in der ZNA implementiert. Durch Verknüpfung der COVID-19-Risikokategorien mit spezifischen Isolations‑, Hygiene- und Personalschutzmaßnahmen wurden alle Bereiche der ZNA neu strukturiert. Retrospektiv erfolgte die statistische Auswertung aller stationären Fälle (*n* = 491) innerhalb eines vierwöchigen Zeitraums.

**Ergebnisse:**

Im Patientenkollektiv wurden *n* = 25 (5,1 %) SARS-CoV-2-positive Fälle identifiziert. Diese verteilten sich prozentual auf die Risikokategorien wie folgt: RK I – bestätigte SARS-CoV-2-Infektion 36 % (*n* = 9), RK II – begründete Verdachtsfälle 32 % (*n* = 8), RK III – differenzialdiagnostische Abklärung 12 % (*n* = 3), RK IV – geringe Wahrscheinlichkeit 8 % (*n* = 2) und RK V – kein Verdacht 12 % (*n* = 3). Bis dato ist keine Transmission der SARS-CoV-2-Infektion bei Mitarbeitern oder Patienten in der ZNA aufgetreten.

**Schlussfolgerung:**

Die Einführung der COVID-19-Risikokategorien ermöglicht die zentrale Steuerung der krankenhaushygienisch relevanten Prozesse einer ZNA im Kontext der COVID-19-Pandemie. Durch eine stetige Reevaluation der Falldefinitionen können lokale Ausbruchssituationen berücksichtigt werden. Die COVID-19-Risikostratifizierung ermöglicht eine strikte Trennung von COVID-19/Non-COVID-19-Notfällen und stellt so die nosokomiale Infektionsprävention für Personal und Patienten sicher.

## Abstract (en)

**Background:**

The COVID-19 pandemic represents a complex challenge for medical staff within emergency departments (ED) of hospitals at all care levels. Beside regular emergency care, rapid detection and isolation of COVID-19 cases are obligatory for prevention of internal viral transmission and efficient medical staff protection.

**Methods:**

In this study a model of risk stratification for suspected SARS-CoV‑2 and COVID-19 cases was developed on the basis of epidemiologic criteria of the Robert-Koch Institute including five risk categories (RC). The model was implemented in a hospital of basic and regular care level. By combination of risk categories with specific isolation, hygienic and personal protection procedures all areas of the ED were restructured. In a retrospective study all inpatient cases (*n* = 491) were re-evaluated during a 4-week interval (26 March–26 April 2020).

**Results:**

In the study population 25 SARS-CoV‑2 positive cases (5.2%) were identified. These cases were categorized according to the risk stratification model as follows: RC I—confirmed SARS-CoV‑2 infection 36% (*n* = 9), RC II—reasonable suspected cases 32% (*n* = 8), RC III—differential diagnostic cases 12% (*n* = 3), RC IV—low probability 8% (*n* = 2) and RC V—no evidence 12% (*n* = 3). No viral transmission was detected during the whole period within medical staff and patients of the ED.

**Conclusions:**

Introduction of COVID-19 risk categories within the ED permits central control of important hygienic processes with respect to SARS-CoV‑2 infection probability. By continuous re-evaluation of case definitions local outbreaks can be used to adapt criteria within the risk categories. Risk stratification of COVID-19 cases allows for a strict separation of COVID-19 and non-COVID-19 emergencies and thus ensures effective infection prevention of medical staff and patients.

Ende Dezember 2019 wurden erstmalig in Wuhan (Hubei, China) mehrere Fälle einer viralen Lungenentzündung festgestellt. Die Sequenzanalysen des viralen Genoms ergaben ein Betacoronavirus aus der Familie der Coronaviridae. Für die durch SARS-CoV‑2 („severe acute coronavirus 2“) verursachte Erkrankung wurde von der WHO die Bezeichnung COVID-19 („coronavirus disease 2019“) eingeführt [1]. Als Folge der raschen Ausbreitung des Erregers erfolgte am 11.03.2020 die Einstufung von COVID-19 als Pandemie.

Der Zentralen Notaufnahme (ZNA) kommt in der Versorgung von COVID-19-Patienten in mehrfacher Hinsicht eine besondere Bedeutung zu [2]. Im Vordergrund steht die primäre Erkennung und Behandlung von akuten Erkrankungen als Folge einer SARS-CoV-2-Infektion. Die ZNA stellt darüber hinaus einen Filter für COVID-19-Patienten für das gesamte Krankenhaus dar, denn deren frühzeitige Identifikation ist eine Grundvoraussetzung für die Einleitung von Schutzmaßnahmen für Mitarbeiter und Patienten. Ein besonderes Augenmerk gilt hierbei den Patienten mit Risikofaktoren (u. a. Alter, Immundefizienz, Lungen‑, Herz‑, Nierenerkrankungen, Malignome). Diese sog. vulnerable Gruppe muss vor nosokomialen Infektionen in allen Bereichen eines Krankenhauses geschützt werden.

Zur Einführung einer strukturierten Lenkung der Patientenströme im Kontext der COVID-19-Pandemie wurde in dieser Arbeit in der ZNA eines Krankenhauses der Grund- und Regelversorgung ein Modell für die Risikostratifizierung von COVID-19-Fällen entwickelt und organisatorisch umgesetzt. Hierbei wurde das Ziel verfolgt, durch die Veränderungen der Prozessabläufe einerseits eine schnellstmögliche optimale Diagnostik und Therapie der COVID-19-Fälle umzusetzen und andererseits den bestmöglichen Infektionsschutz für Mitarbeiter und Patienten zu gewährleisten.

## Methoden

Das Evangelische Krankenhaus Göttingen-Weende ist ein Krankenhaus der Grund- und Regelversorgung und gleichzeitig ein akademisches Lehrkrankenhaus der Universitätsmedizin Göttingen. Das Krankenhaus verfügt über 601 Betten an 3 Standorten mit 15 Fachabteilungen. In der Zentralen Notaufnahme (ZNA) werden pro Jahr ca. 30.000 Notfälle behandelt.

Zur Bewältigung der COVID-19-Pandemie wurden in der ZNA des Krankenhauses zahlreiche Maβnahmen ergriffen mit dem Ziel, die Prozessabläufe in der ZNA zu beschleunigen und den Infektionsschutz für Mitarbeiter und Patienten zu gewährleisten. Dabei wurden die 3 Säulen der Virusbekämpfung [3] des Robert-Koch Institutes (RKI) berücksichtigt:

1. Erhöhung der Behandlungskapazitäten,
2. Schutz der vulnerablen Gruppen,
3. Eindämmung der Ausbreitung.

Hierzu wurde neben einer Änderung der räumlichen Strukturierung der ZNA ein neues Modell für die Risikostratifizierung von SARS-CoV-2-Verdachtsfällen und COVID-19-Patienten implementiert: Das Modell wurde auf der Basis der epidemiologischen Kriterien des RKI entwickelt und um interne Falldefinitionen ergänzt. Es umfasst 5 Risikokategorien (RK):

1. RK I – bestätigte SARS-CoV-2-Infektion,
2. RK II – begründeter Verdacht,
3. RK III – differenzialdiagnostische Abklärung,
4. RK IV – geringe Wahrscheinlichkeit,
5. RK V – kein Verdacht.

Der Behandlungspfad wurde für jede Risikokategorie mit spezifischen Hygienemaßnahmen und definierten Behandlungsorten in der ZNA sowie in der stationären Versorgung verknüpft (Tab. 1).

**Table 1:** 

COVID-19 Risikokategorie	Beschreibung	Kriterien	Hygienemaßnahmen	ZNA Behandlungsort	Stationäre Versorgung
**I**	Bestätigte SARS-CoV-2-Infektion	Nasen-Rachen-Abstrich (extern) RT PCR positiv	ZNA-Mitarbeiter: PSA Vollschutz^a^ Patient: MNS^b^ Scheuer-Wisch-Desinfektion	Raum Kategorie I	COVID-19-Station
**II**	COVID-19 Begründeter Verdacht	Akute respiratorische Symptome oder Fieber *plus* Kontakt zu bestätigtem COVID-19-Fall in den letzten 14 Tagen oder Übernahme aus Pflegeheim/KH mit und ohne Ausbruchsituation	ZNA-Mitarbeiter: PSA Vollschutz Patient: MNS Scheuer-Wischdesinfektion	Raum Kategorie II	COVID-19 Verdachtsstation
**III**	COVID-19 Differenzialdiagnostische Abklärung	Unspezifische Allgemeinsymptome oder Fieber *plus* Tätigkeit in med. Beruf oder Zugehörigkeit Risikogruppe *plus* *Keine* wahrscheinliche Alternativdiagnose	ZNA-Mitarbeiter: PSA Vollschutz Patient: MNS Kontakt-Flächendesinfektion	Raum Kategorie III	COVID-19 Verdachtsstation
**IV**	COVID-19 Geringe Wahrscheinlichkeit	Unspezifische Allgemeinsymptome oder Fieber *plus* Tätigkeit in med. Beruf oder Zugehörigkeit Risikogruppe *plus* *wahrscheinliche Alternativdiagnose*	ZNA-Mitarbeiter: MNS, Face Shield Patient: MNS Kontakt-Flächendesinfektion	Raum Kategorie IV	Vorisolation Barrieremaßnahmen
**V**	COVID-19 Kein Verdacht	Weder anamnestischer noch klinischer Verdacht	ZNA-Mitarbeiter: MNS, Face Shield Patient: MNS Kontakt-Flächendesinfektion	Raum Kategorie V	Vorisolation Barrieremaßnahmen

^a^Persönliche Schutzausrüstung (PSA) Vollschutz Schutzkittel, Handschuhe, FFP2/3-Maske, Visier/Schutzbrille

^b^MNS Mund-Nasen-Schutz

In einer retrospektiven Studie wurde nachfolgend eine Analyse aller stationären Aufnahmen der ZNA durchgeführt zur Beurteilung der Effizienz der getroffenen Maßnahmen und Güte der Risikostratifizierung in der vierwöchigen Hauptphase der COVID-19-Pandemie (26.03. bis 26.04.2020).

Die statistische Analyse erfolgte unter Verwendung von SAS 9.4 und Microsoft Excel mit Bestimmung der absoluten und relativen Häufigkeiten sowie Sensitivität und Spezifität. Für den Gruppenvergleich des Alters wurde der Zweistichproben-t-Test für unabhängige Stichproben eingesetzt. Ein *p*-Wert von <0,05 wurde als signifikant betrachtet.

## Ergebnisse

In der ZNA des Krankenhauses der Grund- und Regelversorgung wurden die im Folgenden genannten Maßnahmen zur Bewältigung der COVID-19-Pandemie umgesetzt.

### Räumliche Strukturveränderungen zur Erhöhung der Behandlungskapazitäten

Für alle Notfallpatienten wurde der Zutritt zur ZNA reglementiert. Im Bereich des Liegendeingangs wurde ein Durchgangszelt errichtet, um wartende Patienten vor Wettereinflüssen zu schützen. Des Weiteren wurden die Räumlichkeiten der ZNA durch Stellwände erweitert. Hierdurch konnte die Schockraum-Computertomographie (CT) in den Isolationsbereich integriert und für die rasche Diagnostik ohne aufwändige Transportmaßnahmen gewonnen werden. Durch die Allokation am Rande des Isolationsbereichs stand die CT dabei jederzeit für alle traumatologischen Fälle zur gezielten CT-Diagnostik unmittelbar zur Verfügung. Gleichzeitig wurde additiv ein mobiles Röntgengerät im CT-Raum platziert, um eine konventionelle Röntgendiagnostik bei Unfallverletzten mit erhöhtem Risiko für COVID-19 zu ermöglichen.

**Figure 1:**
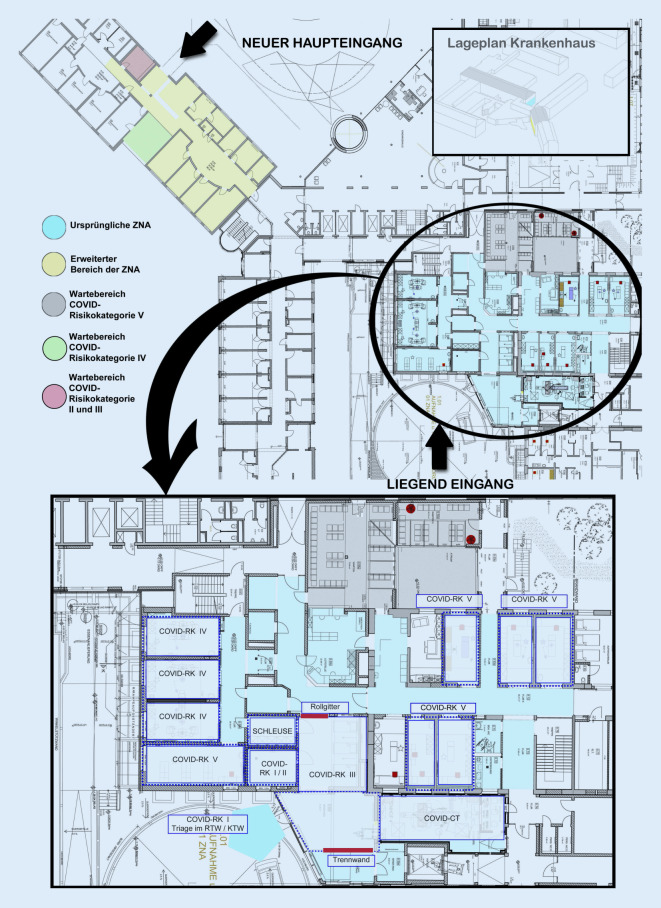

Der Isolationsbereich ist von der restlichen ZNA durch ein Rollgitter und ein Schleusensystem getrennt, welches bereits im Rahmen der Ebola-Epidemie von 2014/15 baulich geschaffen wurde. Darüber hinaus wurde der Haupteingang des Krankenhauses verlegt und alle Nebeneingänge verschlossen, sodass nur ein Eingang für gehfähige Patienten verblieben ist. Daran schließt sich ein durch Plexiglas geschützter Bereich an, der der Patientenbefragung und Administration dient. Angrenzend hieran wurden weitere Räume der ehemaligen Tagesklinik gewonnen, die als Wartezimmer und für die Patientenversorgung genutzt werden. Somit kann die Versorgung von ambulanten Notfallpatienten aller Fachrichtungen in den Räumlichkeiten der erweiterten ZNA durchgeführt werden, ohne dass der Hauptbereich des Krankenhauses betreten werden muss (Abb. 1).

### Implementierung der Risikostratifizierung von SARS-CoV-2-Verdachtsfällen und COVID-19-Patienten

Zur Infektionsprävention und zum Schutz vulnerabler Risikogruppen wurde ein Modell der Risikostratifizierung aller Notfallpatienten in Bezug auf eine SARS-CoV-2-Infektion eingeführt. Auf der Basis der Vorgehensweise des RKI zur SARS-CoV-2-Verdachtsabklärung wurde dazu eine Einteilung in 5 Risikokategorien anhand definierter Kriterien entwickelt (Tab. 1). Diese umfassen Risikokategorie I (bestätigte SARS-CoV-2-Infektion), Risikokategorie II (begründeter Verdachtsfall), Risikokategorie III (differenzialdiagnostische Abklärung), Risikokategorie IV (geringe Wahrscheinlichkeit) und Risikokategorie V (kein Verdacht). Diese Einteilung berücksichtigt dabei über das RKI-Schema hinausgehend tagesaktuell bekannte Ausbruchsituationen in den örtlichen Pflegeheimen und naheliegenden Krankenhäusern (Risikokategorie II). Darüber hinaus werden die vulnerablen Risikogruppen und weitere unspezifische Symptome in der Kategorisierung berücksichtigt (Risikokategorie III und IV).

**Figure 2:**
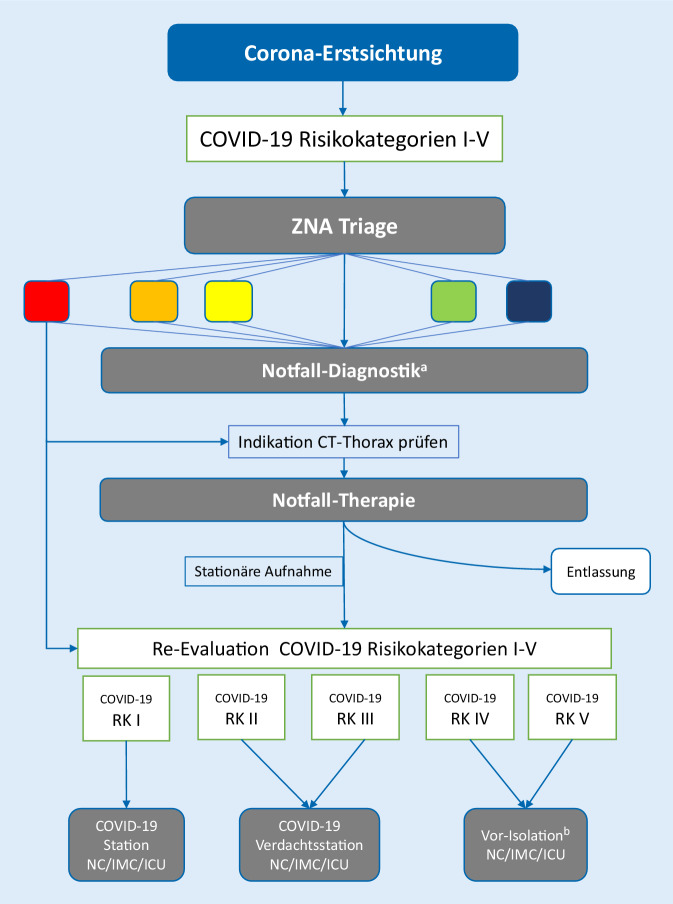

Auf der Basis dieses Stufenschemas wurde für alle Notfallpatienten ein strukturierter Behandlungspfad festgelegt (Abb. 2): Alle Notfallpatienten erhalten zunächst eine COVID-19-Erstsichtung durch den zuständigen Arzt und Pflegekraft der ZNA nach dem Vier-Augen-Prinzip. Jeder Patient erhält demgemäß einen COVID-19-Risikostatus. Zur Feststellung der Behandlungsdringlichkeit wird nachfolgend eine Triage nach dem Manchester Triage System (MTS) [4] durchgeführt. Gemäß Risikostatus erfolgt dann eine Zuweisung des primären, entsprechend gekennzeichneten Behandlungs- und Isolationsortes in der ZNA. Die Risikokategorisierung ist darüber hinaus mit den spezifischen Hygieneschutzmaßnahmen verknüpft (Tab. 1). Bei der Versorgung von Patienten der Kategorien COVID-19-Risikostatus I–III trägt das Personal Schutzkittel, FFP2-Maske, Schutzbrille und Handschuhe. Bei der Versorgung von Patienten mit dem COVID-19-Risikostatus IV und V tragen die Mitarbeiter einen Mund-Nasen-Schutz sowie ein Mund-Gesichts-Schutz (sog. „face shield“) in Anlehnung an die Empfehlung der Deutschen Gesellschaft für Allgemeinmedizin (DEGAM; [5]).

Zur Notfalldiagnostik bei COVID-19-Risikostatus II–IV wurde ein standardisiertes Vorgehen implementiert: Neben der Bestimmung der Vitalparameter (RR, Puls, S_p_O_2_) erfolgt eine Labordiagnostik (Blutbild, Differenzialblutbild, Kreatinin, Elektrolyte, LDH, PCT, CRP, aBGA). Bei allen Patienten erfolgt ein Nasen-Rachen-Abstrich und eine Asservierung von Rachenspülwasser für die PCR-Diagnostik auf SARS-CoV‑2 und ggf. für die Durchführung eines Influenza-Schnelltests (nur im Zeitraum der Influenza-Epidemie). Zur bildgebenden Diagnostik wurde als Standard die Thorax-Sonographie als fester Bestandteil der Diagnostik basierend auf den Empfehlungen von Volpicelli et al. [6] eingeführt. Pleuraergüsse, Pneumothorax oder die Zeichen der kardialen Insuffizienz als Ursache der respiratorischen Insuffizienz sollen hierdurch primär erkannt und auf aufwändige Patiententransporte in die Röntgenabteilung verzichtet werden. Bei begründeten Verdachtsfällen (COVID-Risikokategorie II) oder bei hoher klinischer Wahrscheinlichkeit für COVID-19, Nachweis einer Lymphopenie und negativem Procalcitonin wird bei indizierter stationärer Behandlung eine Low-dose-CT-Untersuchung des Thorax durchgeführt [7–9] und gemäß CO-RADS-Kategorien [10] klassifiziert. Nach Durchführung der Notfalldiagnostik erfolgt bei stationär zu behandelnden Patienten eine Reevaluation der COVID-19-Risikostatus basierend auf den Untersuchungsergebnissen und nachfolgend die Auswahl der jeweiligen Zielstation gemäß Kategorisierung (Tab. 1).

### Retrospektive Analyse der Behandlungsdaten stationärer Notfallpatienten

**Figure 3:**
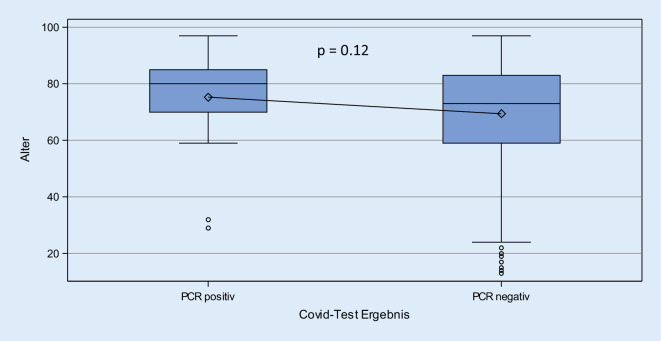

**Figure 4:**
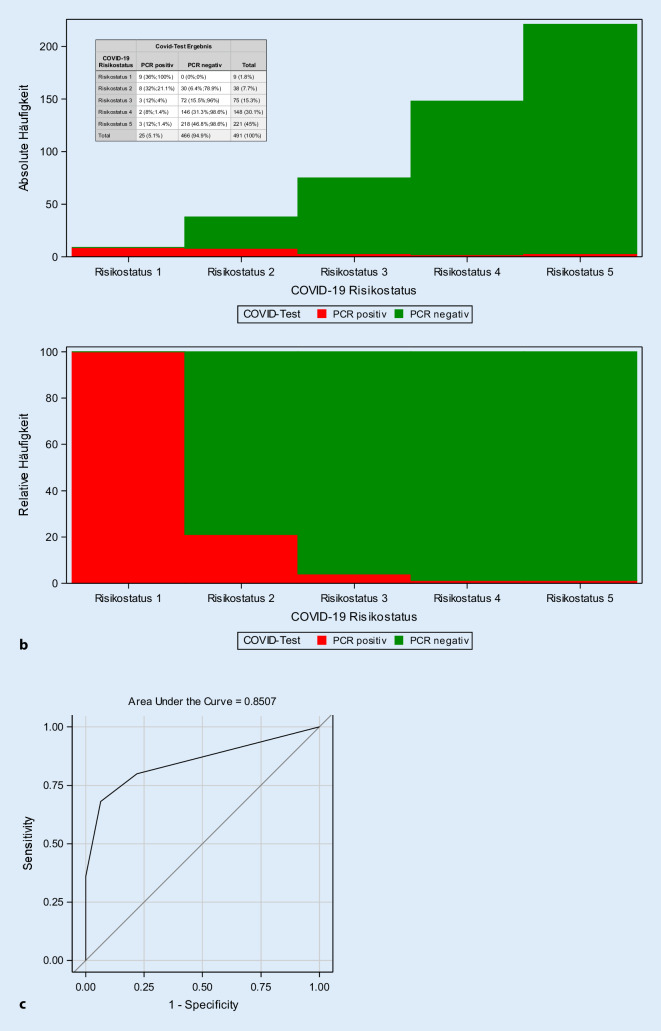

Im Zeitraum vom 26.03. bis 26.04.2020 wurden 491 Patienten über die ZNA stationär aufgenommen. In diesem Patientenkollektiv wurden *n* = 25 (5,1 %) SARS-CoV-2-Infektionen detektiert. Es bestand kein signifikanter Unterschied zwischen dem mittleren Alter der SARS-CoV-2-positiven und dem Kollektiv der SARS-CoV-2-negativen Fälle (*p* = 0,12; Abb. 3). Im Gesamtkollektiv wurden 1,8 % der Fälle in den COVID-Risikostatus I klassifiziert, davon waren 100 % in der SARS-CoV-2-PCR-Diagnostik positiv. Im COVID-19-Risikostatus II wurden 7,7 % der Fälle klassifiziert (*n* = 38), hiervon wurden 21,1 % (*n* = 8) als positive Fälle identifiziert. 15,3 % der Fälle (*n* = 75) wurden dem Risikostatus III zugeordnet; hierbei konnten nur 4 % (*n* = 3) positive Fälle detektiert werden. In den Risikokategorien IV und V mit 148 bzw. 221 Fällen fanden sich nur jeweils 1,4 % positive SARS-CoV-2-Infektionen (Abb. 4). Betrachtet man den COVID-Risikostatus I–IV als ein positives Testergebnis und den COVID-Risiko-Status V (kein Verdacht) als ein negatives Testergebnis, so besitzt der Test eine Sensitivität von 88,0 % und eine Spezifität von 46,8 %. Die in den COVID-19-Algorithmus integrierte CT-Diagnostik wurde in *n* = 58 (12,9 %) Fällen eingesetzt; dabei wurde in einem Viertel der Fälle eine pulmonale Manifestation der SARS-CoV-2-Infektion im Sinne eines hochgradigen Verdachts (CO-RADS 4, *n* = 8) bzw. COVID-19-typischer Befunde (CO-RADS 5, *n* = 7) detektiert. Im gesamten Zeitraum konnte keine interne Transmission einer SARS-CoV-2-Infektion bei Patienten oder Mitarbeitern der ZNA nachgewiesen werden.

## Diskussion

Die frühzeitige Erkennung aller Patienten, die mit SARS-SoV‑2 infiziert sind, stellt eine große Herausforderung für eine ZNA dar, wie die Arbeiten von Cho et al. aus dem Jahr 2016 anhand eines Ausbruchs von MERS-CoV („middle east respiratory syndrome-related coronavirus“) durch lediglich einen erkrankten Patienten in der ZNA belegen [11]. Auch kann ein Krankheitsausbruch unter den Mitarbeitern weitere Patienten gefährden und zu einem erheblichen Ausfall hoch qualifizierten Personals führen [12].

Für die Diagnostik der floriden SARS-CoV-2-Infektion wird als Goldstandard der molekularbiologische Virusnachweis mittels Reverse-Transkriptase-Polymerase-Kettenreaktion (RT-PCR; [13–15]) eingesetzt. Dieser Test ist jedoch in der Regel zeitintensiv und auch nicht jederzeit und überall verfügbar. Die aktuelle Entwicklung von Point-of-Care und automatisierten Testverfahren [16, 17] hat hier zu einer erheblichen Beschleunigung der diagnostischen Prozessabläufe geführt. Dennoch gelingt der Nachweis der SARS-CoV-2-Infektion nur in der replikativen Phase der viralen Infektion, da die Kopienzahl im Verlauf der SARS-CoV-2-Infektion sinkt [18]. Auch können durch die RT-PCR nicht alle Erkrankten identifiziert werden [12], wie die klinische Studie bei 138 hospitalisierten COVID-19-Patienten in Wuhan gezeigt hat. In einer weiteren Studie wurde die Sensitivität der RT-PCR sogar mit 71 % signifikant geringer bestimmt als pathologische Veränderungen, die mittels CT-Untersuchung der Lunge detektiert wurden [19].

Neben den molekularbiologischen Testverfahren stehen mittlerweile auch serologische Verfahren zur Antikörperdiagnostik gegen SARS-CoV‑2 zur Verfügung. Trotz einer hohen Spezifität liegt die Sensitivität dieser Verfahren, u. a. bedingt durch Kreuzreaktivität mit anderen Coronaviren, IgM-Rheumafaktoren, jedoch nur zwischen 70 und 90 % [20, 21]. Darüber hinaus sind Antikörpertests gerade in der Frühphase der Infektion nicht zur Detektion der COVID-19-Erkrankung in der Notaufnahme geeignet, da eine Serokonversion in der Regel erst in der zweiten Woche der Infektion zu erwarten ist [15].

Die offiziellen Falldefinitionen des RKI sind für die umfassende Erkennung der an COVID-19 erkrankten Patienten in der Notaufnahme nicht ausreichend. Sie stellen in erster Linie eine Grundlage dar, gezielte Testungen auf COVID-19 durch eine RT-PCR zu initiieren und gleichzeitig die Gesundheitsbehörden bei begründeten Verdachtsfällen zu informieren. Frühzeitig wurde hier in dieser Arbeit somit ein Modell für eine eigene Risikostratifizierung entwickelt und umgesetzt, die eine höhere Sensitivität in der Erkennung der Verdachtsfälle erreicht (88 %). Ein gleicher Ansatz findet sich auch in den Arbeiten von Wee Le et al. [22] aus Singapur. Hier konnte gezeigt werden, dass die zusätzlichen Falldefinitionen zu einer Steigerung der Sensitivität von 49 auf 84 % in der Identifikation von COVID-19-Fällen geführt hat.

Als Erweiterung hierzu wurde in dieser Arbeit die COVID-Risikokategorie I–V eingeführt. Hierdurch soll erreicht werden, dass besondere Risikogruppen mit chronischen Erkrankungen (COVID-Risikostatus IV, wahrscheinliche Alternativdiagnose) nicht regelhaft mit Patienten zusammen behandelt werden, die ein höheres Risiko (COVID-Risikostatus II und III) haben, an COVID-19 erkrankt zu sein. Diese Patienten stellen zum einen die Risikopersonen für COVID-19 dar (Bewohner aus Pflegeheimen, chronisch Lungenerkrankte etc.), gleichzeitig benötigen sie zur Vermeidung einer nosokomialen Transmission jedoch besondere Isolationsmaßnahmen (Schutz der vulnerablen Gruppen).

Über die Falldefinitionen als Grundlage für die COVID-Risikokategorien können die hieran gekoppelten Maßnahmen in Abhängigkeit der lokalen, regionalen und überregionalen Lage risikobasiert gesteuert und dementsprechend die Sensitivität und die Spezifität des Verfahrens verändert werden. Wee LE et al. [22] empfehlen in ihrer Arbeit eine balancierte Falldefinition, denn je mehr Patienten durch die Falldefinitionen die COVID-Risikostatus I–IV erhalten, desto höher wird der logistische Aufwand für die ZNA. Zur Bewältigung der gesteigerten Anforderungen wurden, wie in den Arbeiten aus Singapur ebenfalls beschrieben, die räumlichen Ressourcen der ZNA daher erweitert [22].

Darüber hinaus wurde insbesondere für Patienten mit der COVID-Risikostatus II, III und IV ein Flussschema zur Optimierung der Abläufe in der ZNA erarbeitet. Dieses Vorgehen wurde in ähnlicher Weise in sog. Fieber Kliniken in Wuhan (China; [7]), aber auch in Universitätsklinika in Deutschland [8, 23] implementiert. Ziel dieser Maßnahmen ist es, eine Überlastung der ZNA zu vermeiden, um entsprechende Schutz- und Isolationsmaßnahmen aufrechthalten zu können [2]. Zusätzlich wird hierdurch die Gefahr vermindert, vital bedrohliche Erkrankungen verspätet zu behandeln. So konnten Tam CF et al. bereits negative Einflüsse der COVID-19-Pandemie auf die Versorgung von ST-Hebungsinfarkten in Hong Kong nachweisen [24].

Bei der retrospektiven Untersuchung des eigenen Patientenguts von 25 Patienten mit COVID-19-gesicherter Erkrankung konnte hier gezeigt werden, dass 3 (12 %) Patienten primär nicht in der ZNA erkannt wurden (Abb. 4). Auch diese Ergebnisse decken sich mit den Arbeiten von Wee LE et al. [22], in der 16 % der COVID-19-positiven Patienten nicht in der Notaufnahme identifiziert werden konnten. Als Konsequenz hieraus werden auch COVID-Risikokategorie‑V Fälle bis zum Erhalt des PCR-Ergebnisses vorisoliert, und es wurde der Personalschutz in der ZNA in Anlehnung an die Empfehlung der DEGAM [5] um das Tragen eines Face Shields erweitert.

Durch die Einführung des COVID-Risikostatus basierten Hygieneplans und die Durchführung der hier beschriebenen Maßnahmen zum Personalschutz, zur Isolation der Patienten und zur hygienischen Aufbereitung der Arbeitsbereiche konnte dementsprechend erreicht werden, dass im Rahmen des umfassenden Personalscreenings kein einziger positiver Befund in der SARS-CoV‑2 RT-PCR im Rachenspülwasser nachweisbar war. Auch wurde bis dato keine Übertragung auf andere Patienten in der ZNA nachgewiesen.

Zusammenfassend ermöglicht die Einführung des hier beschriebenen Modells der Risikostratifizierung von SARS-CoV-2-Verdachts- und COVID-19-Fällen eine zentrale Steuerung der krankenhaushygienisch relevanten Prozesse einer ZNA im Kontext der COVID-19-Pandemie. Durch eine stetige Reevaluation der Falldefinitionen im Modell können darüber hinaus kurzfristig lokale Ausbruchssituationen in den Risikokategorien berücksichtigt werden. Die COVID-19-Risikostratifizierung ermöglicht auf diese Weise eine strikte Trennung von COVID-19- und Non-COVID-19-Notfällen und stellt so die nosokomiale Infektionsprävention sowohl für Mitarbeiter als auch für Patienten in der ZNA sicher.

## Fazit für die Praxis

- In der ZNA gelten für die Bewältigung der Anforderungen im Kontext der COVID-19-Pandemie die 3 Säulen der Virusbekämpfung des Robert Koch-Instituts (RKI): Erhöhung der Versorgungsmöglichkeiten, Eindämmung der Ausbreitung und Schutz der vulnerablen Gruppen. Durch die Einführung eines Modells zur COVID-19-Risikostratifizierung können alle 3 Säulen adressiert werden.
- Zur Optimierung der notfallmedizinischen Versorgungsmöglichkeiten in Krankenhäusern aller Notfallversorgungsstufen müssen die räumlichen Ressourcen erweitert und die Behandlungsabläufe neu strukturiert werden, um einer Überlastung der ZNA entgegenzuwirken und die Infektionsprävention für Mitarbeiter und Patienten sicherzustellen.
- Die Kriterien für die Bestimmung der COVID-19-Risikokategorie müssen ständig reevaluiert werden, um auf die Änderung der lokalen, regionalen und überregionalen Lage der COVID-19-Pandemie kurzfristig reagieren zu können.
- Eine Risikostratifizierung für SARS-CoV-2-Verdachts- und COVID-19-Fälle in der Zentralen Notaufnahme jeder Notfallversorgungsstufe ermöglicht eine wirksame Infektionsprävention für Mitarbeiter und Patienten in der ZNA durch strikte Trennung von COVID-19- und Non-COVID-19-Notfällen.
